# CMTM5-v1 inhibits cell proliferation and migration by downregulating oncogenic EGFR signaling in prostate cancer cells

**DOI:** 10.7150/jca.42314

**Published:** 2020-04-06

**Authors:** Yeqing Yuan, Zhengzuo Sheng, Zhenhua Liu, Xiaowei Zhang, Yunbei Xiao, Jing Xie, Yixiang Zhang, Tao Xu

**Affiliations:** 1 Department of Urology, Shenzhen People's Hospital, The Second Clinical Medical College of Jinan University, The First Affiliated Hospital of Southern University of Science and Technology, Shenzhen, 518020, China; 2 Department of Thoracic Surgery, Fu Xing Hospital, Capital Medical University, Beijing, 100038, China; 3 Department of Urology, Beijing Jishuitan Hospital, Beijing, 100096, China; 4 Department of Urology, Peking University People's Hospital, Beijing, 100044, China; 5 Department of Urology, The First Affiliated Hospital of Wenzhou Medical University, Wenzhou 325000, China

**Keywords:** CMTM5, tumor suppressor gene, castration-resistant prostate cancer, EGFR, tyrosine kinase inhibitors, Gefitinib

## Abstract

Anomalous epidermal growth factor receptor (EGFR) signaling plays an important role in the progression of prostate cancer (PCa) and the transformation to castration-resistant PCa (CRPC). A novel tumor suppressor CKLF-like MARVEL transmembrane domain-containing member 5(CMTM5) has a MARVEL domain and may regulate transmembrane signaling. Thus, we postulated that CMTM5 could regulate EGFR and its downstream molecules to affect the biological behaviors of PCa cells. In this study, we found that CMTM5 was expressed in benign prostatic hyperplasia (BPH) tissues but was undetectable in PCa cells. However, the EGFR was upregulated in PCa cells, especially in two metastatic CRPC cell lines, PC3 and DU145. Furthermore, ectopic expression of CMTM5-v1 suppressed cell proliferation and migration and p-EGFR levels. Further investigation revealed that restoration of CMTM5-v1 inhibited not only EGF-mediated proliferation but also chemotactic migration by EGF in PC3 and DU145 cells. Moreover, mechanistic studies showed that CMTM5-v1 attenuated EGF-induced receptor signaling by repressing EGFR and Akt phosphorylation in PCa cells, which were essential for malignant features. Finally, CMTM5-v1can promote the sensitivity of PC3 cells to Gefetinib, a tyrosine kinase inhibitor (TKI) targeting the EGFR. These observations indicate that CMTM5-v1 suppressed PCa cells through EGFR signaling. The loss of CMTM5 may participate in the progression of PCa resulting from deregulated EGFR, and CMTM5 might be associated with the efficacy of TKIs in terms of their potent inhibition of EGFR and human epidermal growth factor-2 (HER2) activation.

## Introduction

Prostate cancer (PCa) is one of the most common malignant neoplasms of the genitourinary system among men and is the second leading cause of male cancer death in the United States 1. The incidence of PCa in China has increased rapidly in recent years 2 . The recommended treatment for patients with progressive carcinoma of the prostate is hormone ablation achieved by surgical or pharmacological castration 3. Most patients initially respond well to this treatment but inevitably relapse and develop an incurable castration-resistant condition that results in death within a few months 4. The molecular mechanisms of PCa development, progression and hormone-independence, which are of the utmost importance for discovering a curative therapy, are not yet well understood. Alterations of several pathways involving growth factor receptors may be implicated in this complicated and heterogeneous process 5, 6. In particular, increased or constitutively activated expression of the epidermal growth factor receptor (EGFR) accounts for a significant proportion of PCa patients and is strongly associated with a high relapse rate, poor prognosis and progression to castration-resistant status 7-10. Previous studies have indicated that EGFR and its downstream pathways could enhance androgen receptor (AR) activity and responses to low levels of androgen 11, and increased EGFR expression (which escapes androgen regulation) is a potential molecular switch for castration-resistant prostate cancer (CRPC) 12. Therefore, the EGFR signaling pathway could be an attractive target for cancer therapy.

Anti-EGFR molecule-targeted drugs, such as antibodies against the ligand-binding domain and small molecule tyrosine kinase inhibitors (TKIs), have been approved by the Food and Drug Administration for the treatment of several tumors or are under evaluation in clinical trials. Gefitinib is one of the clinically used TKIs and has been successfully applied to EGFR-driven tumors in the clinic, especially for non-small cell lung cancer (NSCLC) patients with EGFR mutations on exon 18 or exon 21 within the kinase domain 13, 14. However, CRPC does not respond to Gefitinib 15, 16, which indicates that the mechanisms of EGFR regulation in CRPC are more intricate and deserve to be further explored.

CKLF-like MARVEL transmembrance domain containing (CMTM) is a novel family of proteins consisting of nine members, Chemokine-like factor (CKLF) and CMTM1~CMTM8 17, 18. CMTM5 is a 223-amino-acid multi-pass membrane protein and is structurally similar to the transmembrane 4 superfamily (TM4SF) 18. Members of TM4SF include the typical tetraspanin, myelin and lymphocyte protein (MAL) and other related proteins for vesicle trafficking and membrane-linked (MARVEL) domain-containing proteins, which are involved in multiple cancers 19-23. Located on chromosome 14 at position 14q11.2, an important locus associated with the pathogenesis of multiple carcinomas 24-27, CMTM5 also exhibits potential tumor suppressor activities, similar to TM4SF. A previous study indicated that CMTM5-v1, the major expressed mRNA splicing variant of CMTM5, contains 156 amino acids and is broadly expressed in human fetal and adult tissues but is frequently epigenetically silenced by promoter methylation in cells derived from various cancers 28. The role of CMTM5 has been investigated in many human cancer types, such as cervical, pancreatic, ovarian, hepatocellular carcinoma and leukemia 29-34.

Our previous study revealed that CMTM5 expression is significantly reduced or undetectable in PCa tissues and several cancer cells compared with BPH tissues. The decreased expression of CMTM5 closely correlates with high Gleason scores. Furthermore, ectopic ectopic expression of CMTM5-v1 in DU145 cells led to significant inhibition of cell proliferation and migration compared with the control. Restoration of CMTM5 significantly suppressed tumor growth *in vivo* 35. The potential mechanism of CMTM5 action on PCa cells is not clear. It might involve membrane protein sorting and trafficking due to its predicted MALVEL domain, which is part of the machinery of membrane apposition events 36, such as EGFR signaling. However, whether CMTM5 regulates EGFR as part of CRPC suppression has not yet been explored. In the current study, we further explored the functional role of CMTM5 in PCa and investigated the potential mechanisms of CMTM5 in EGF-mediated receptor signaling inhibition of PCa cells, which may represent a new therapeutic strategy for EGFR-targeted treatment.

## Materials and Methods

### Antibodies, chemicals and tissue samples

A polyclonal antibody against CMTM5 (epitope mapping at the C-terminus, capable of detecting CMTM5 isoforms 1-5 of human origin) was given as a gift from Prof. Wenling Han (Peking University Center for Human Disease Genomics, Beijing, China). Monoclonal antibodies against EGFR, p-EGFR (Tyr1173), protein kinase B (Akt), p-Akt (Ser473), human epidermal growth factor receptor-2 (HER2) and p-HER2 (Tyr1248) were purchased from Cell Signaling Technology (Beverly, MA, USA). Horseradish peroxidase (HRP)-conjugated monoclonal antibody against glyceraldehyde 3-phosphate dehydrogenase (GAPDH) was obtained from Kang Chen Biotech (Shanghai, China). Recombinant human EGF was purchased from Peprotech (Rocky Hill, NJ, USA). Gefitinib (ZD1839) was purchased from Selleck Chemicals (Houston, TX, USA). Human benign prostatic hyperplasia (BPH) tissues were obtained from patients undergoing transurethral resection of the prostate at the Peking University People's Hospital with the patients' consent and institutional ethics approval.

### Cell lines and cell culture

Two human metastatic androgen-independent cell lines DU145 and PC3, another androgen-independent cell line 22Rv1 and the androgen-dependent cell line LNCaP clone FGC were obtained from the American Type Culture Collection (ATCC, Manassas, VA, USA). Another strain of LNCaP was purchased from the Cell Resource Center of Peking Union Medical College (Beijing, China). The cells were cultured in modified RPMI-1640 medium (Hyclone, Logan, UT, USA) containing 10% fetal bovine serum (FBS, Hyclone, Logan, UT, USA) supplemented with penicillin (100 U/ml) and streptomycin (100 µg/ml) (Gibco, Grand Island, NY, USA). The cell cultures were maintained at 37ºC in an atmosphere supplemented with 5% CO_2_.

### Cell transfection

Transfection of PC3 and DU145 cells was performed using DNAfect transfection reagent (CW bio, Beijing, China), and FuGENE® HD transfection reagent (Promega, Madison, WI, USA) was used for 22Rv1. pCDNA3.1-myc-hisB (abbreviated as pCDB) and pCDB-CMTM5-v1 plasmids, which were kindly provided by Prof. Wenling Han, were used for transient transfection. Transfection efficiency was monitored by pEGFP-N1 plasmid. Cells with greater than 75% transfection efficiency were used for further experiments.

### Colony formation assay

The assay was performed as described previously 28. Briefly, DU145 and 22Rv1 cells were transfected. The cells were plated 48 h post-transfection into a 6-well plate at a density of 1×10^4^ cells per well with G418 (800 μg/ml and 600 μg/ml, respectively) added. The selective medium was replaced every 3 days. On day 15, G418-resistant colonies were fixed with 2% paraformaldehyde in phosphate-buffered saline (PFA/PBS). After crystal violet staining, colonies with ≥50 cells were counted under a light microscope. Each group was assayed in three wells, and experiment was repeated in triplicate.

### Cell proliferation assays

Cell proliferation was analyzed using the Vybrant® MTT Cell Proliferation Assay Kit (Life technologies, Eugene, OR, USA). Briefly, transfected cells were plated into 96-well plates at a density of 2×10^3^ cells per well and then cultured at 37ºC. At indicated time points, 10 μl of MTT solution was added into each well, and the cells were incubated at 37ºC in a humidified atmosphere containing 5% CO_2_. Four hours later, the cells were lysed using 100 µL of the SDS-HCl solution, and their absorbance was measured at 570 nm to calculate the relative number of viable cells. Results were obtained from three independent experiments in triplicate.

### Cell migration assay

Cell migration assays were performed in 24-well Transwell chambers (Costar, Cambridge, MA, USA). Briefly, transfected cells were serum-starved for 20 h, placed in the upper chamber and allowed to migrate at 37ºC. The chemoattractant in the lower chamber was the serum-free medium plus 20 ng/ml EGF or 10% FBS in different experiments. The suspended media in the lower chamber was removed 8-12 h later. The cells that had invaded the lower side of the filter were fixed with 4% PFA and stained. The cells that passed through the pores into the lower chamber were counted under a phase-contrast microscope. Triplicate wells were performed in each assay, and the experiment was repeated three times.

### Protein extraction and western blot

The whole-cell extracts were obtained by lysing cells with RIPA solution. Protein concentrations were determined using the bicinchoninic acid (BCA) method (Thermo, Rockford, IL, USA). Total protein (30 μg /lane) was separated by 12% SDS-PAGE and subsequently transferred onto nitrocellulose membranes (Millipore, Bedford, MA, USA). After blocking with 5% skim milk in Tris-buffered saline (pH 7.6) (TBS) at room temperature for 1 h, the membranes were incubated overnight at 4ºC with the primary antibodies. After being incubated with the respective second antibodies (Bioss, Beijing, China), the immune complexes were detected using SuperSignal West Pico Chemiluminescent Substrate (Thermo, Rockford, IL, USA).

### Statistical analysis

All the data are presented as the means and standard errors. The Student T test was used to analyze the significance between two groups. All calculations were performed using the SPSS statistical software package. A *P* value <0.05 was considered significant.

## Results

### CMTM5 and EGFR expression in BPH tissues and PCa cell lines

**Figure 1:**
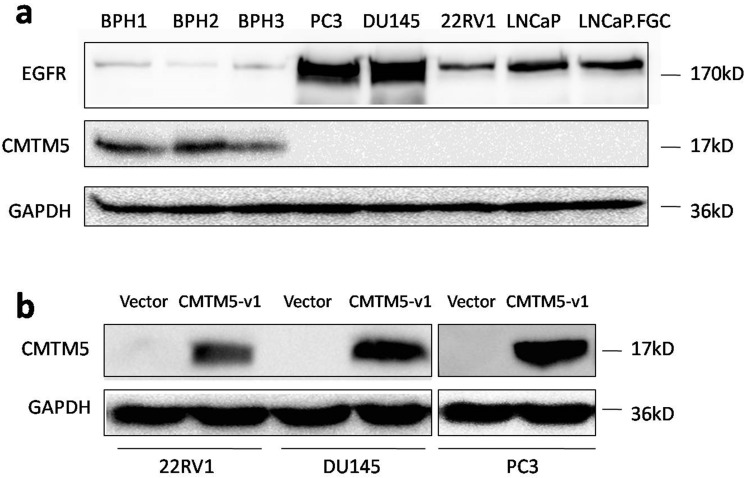
** Expression patterns of CMTM5 and EGFR in PCa. (a)** The endogenous expression patterns of CMTM5 and EGFR in BPH tissues and five PCa cell lines were observed by western blot. **(b)** Forty-eight hours after transfection with empty vector or CMTM5-v1 plasmid, CMTM5 expression in PC3, DU145 and 22Rv1 cells was detected by western blot.

Previously, we showed that CMTM5 is markedly expressed in most of the BPH tissues and is frequently downregulated in PCa tissues, and its expression is negatively correlated with the Gleason score 37. In the current study, the relative expression levels of CMTM5 and EGFR in different PCa cells and BPH tissues were determined by western blot. CMTM5 was expressed in BPH tissues but was undetectable in all five PCa cell lines, and EGFR expression in these cells was much greater than in normal tissues. Furthermore, the androgen-independent cell lines PC3 and DU145 had higher levels of EGFR expression compared to another androgen-independent 22Rv1 cells and androgen-dependent LNCaP cells (Fig.1A). After transfection with the plasmid encoding pCDB-CMTM5-v1, CMTM5 protein expression markedly increased, as assessed by western blotting. In contrast, there was no change in CMTM5 expression in the cells that were transfected with the empty vector (pCDB) (Fig. 1B).

### CMTM5-v1 exerts tumor-suppressive functions in EGFR-overexpressed DU145 cells

**Figure 2:**
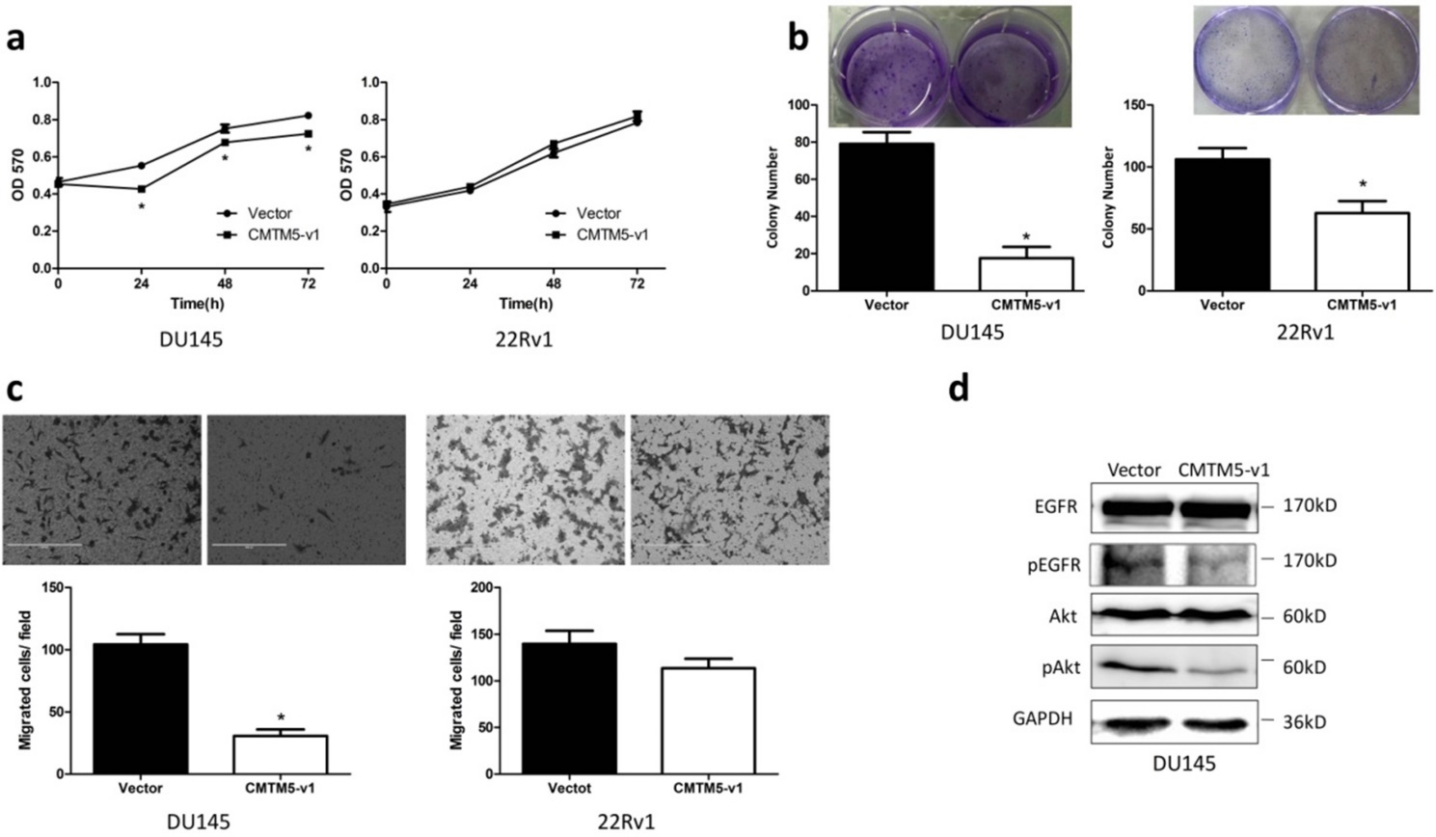
** Effects of CMTM5-v1 on the proliferation and migration of DU145 and 22Rv1 cells.** (a) Twenty-four hours after transfection, cells were plated into 96-well plates and then cultured in normal growth medium. At indicated times, cell proliferation was observed using the MTT assay. The results are expressed as the means ± SEM of three independent experiments. (b) Fifteen days after G418 selection, the effect of CMTM5-v1 on colony-forming capacity was measured by counting the number of colonies ≥50 cells. Bars represent the means ± SEM of three independent experiments (*P<0.05). (c) The metastatic potential was determined using a transwell migration assay with medium plus 10% FBS in the bottom chambers. The graph indicates the means ± SEM of the number of cells per three random fields (magnification, x200) counted from three independent experiments (*P<0.05). (d) Forty-eight hours after transfection, DU145 cells were lysed and used to detect the indicated proteins by western blot.

**Figure 3:**
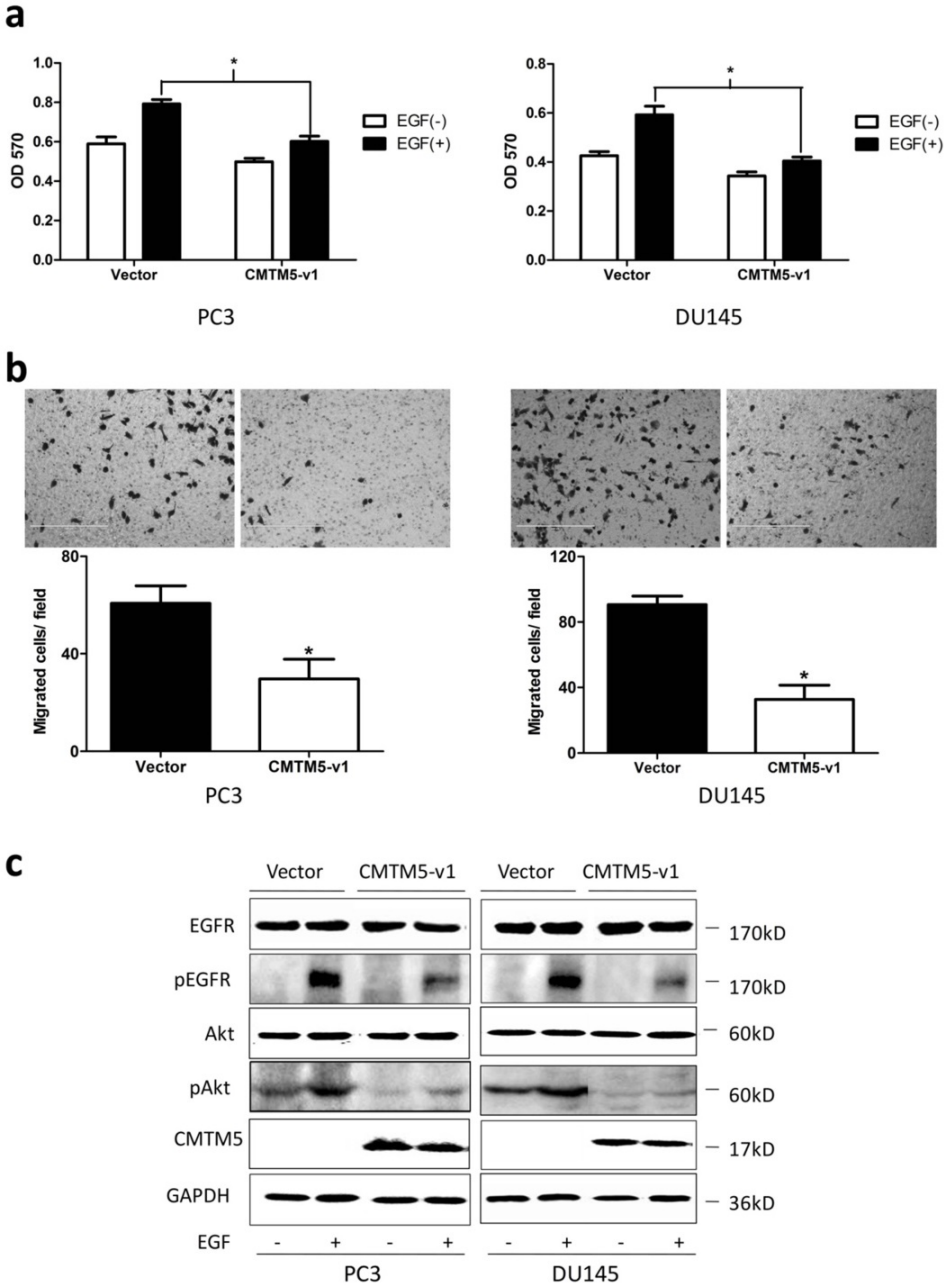
** Effects of CMTM5-v1 on EGF-induced cell growth, migration and EGF-triggered signaling in PC3 and DU145 cells.** (a) Twenty-four hours after transfection, cells were plated into 96-well plates and cultured in serum-free medium overnight. The medium was then switched to RPMI-1640 containing 1% FBS in the presence or absence of 20 ng/ml EGF for 48 h. The MTT assay was performed to analyze cell proliferation. Data represent the means ± SEM of the OD570 values of three independent experiments (*P<0.05). (b**)** The cell migration capacity under EGF-induced chemotaxis was detected in a transwell assay with serum-free medium plus 20 ng/ml EGF in the bottom chambers. Data represent the means ± SEM of cells from three random fields under the microscope, and the experiment was repeated three times. (*P<0.05. Magnification ×200). (c) Transfected cells were serum-starved overnight and then treated with EGF (20 ng/ml) for 5 minutes, and whole-cell lysates were immunoblotted with the indicated antibodies.

Collectively, our results on CMTM5 indicate that it is a potential PCa tumor suppressor gene. To further identify the tumor suppressive capacity of CMTM5 in other CRPC cells, we detected the proliferation and migration properties of DU145 and 22Rv1 cells after transfection. As shown in Fig. 2A, the MTT assay indicated that the proliferation of DU145 cells was significantly inhibited by CMTM5-v1 at all time points. There was no significant effect within the limited observation time for 22Rv1 cells, although the absorbance in CMTM5-v1-transfected cells was less than the control (empty vector). In addition, the colony-forming capacities were significantly weakened by CMTM5-v1 in both cell lines (Fig. 2B). The migration assay indicated that CMTM5-v1- expressing DU145 cells displayed lower transmembrane migration activity than the controls, as shown by a significant decrease in the number of migrated cells. However, there was no significant difference in the 22Rv1 cells (Fig. 2C). Consideration the significantly lower EGFR expression in 22Rv1 compared to DU145, we think that the tumor suppressive activities of CMTM5-v1 in EGFR- overexpressed cells could be more effective. To investigate whether the molecular mechanism of CMTM5-v1 in EGFR-driven metastatic CRPC cells is related to EGFR siganling, we used western blot to detect its expression and phosphorylation. As shown in Fig. 3D, CMTM5-v1 had no effect on total EGFR expression in DU145 cells, but it reduced the phosphorylated EGFR^Try1173^ (p-EGFR^ Try1173^) levels, which represent EGFR signaling activity. We also determined the level of Akt activation, a main downstream pathway initiated by EGFR activation. p-Akt was markedly reduced when CMTM5-v1 was restored. These observations suggest that CMTM5-v1 may regulate EGFR/Akt signaling during tumor pathogenesis and progression.

### CMTM5-v1 inhibits EGF-induced cell proliferation in PCa cells

To investigate whether CMTM5-v1 suppressed the proliferation properties of PCa cells under the induction of EGF, PC3 and DU145 cells were transiently transfected with CMTM5-v1 plasmids and were maintained in culture media supplemented with 1% FBS in the presence or absence of 20 ng/ml EGF. We found that the introduction of CMTM5-v1 resulted in a marked decrease in EGF-induced cell growth. As shown in Fig. 3A, the MTT assay revealed that cell proliferation was significantly increased by EGF in PC3 and DU145 cells transfected with empty vector. However, in the two cell lines transfected with CMTM5-v1, the presence or absence of EGF had no effect, and the effect of EGF on cell proliferation was significantly lower compared to the control cells.

### CMTM5-v1 reduces the chemotactic migration capacity of EGF

To further determine whether the effects of CMTM5 on migration in PCa cells were related to EGF-induced signaling, we utilized a transwell chamber assay to examine the chemotactic migration of PC3 and DU 145 transfectants in response to EGF. As shown in Fig. 3B, the movement of CMTM5-v1- transfected cells was markedly reduced compared with that of vector-transfected cells, indicating that the restoration of CMTM5-v1 could significantly inhibit the PC3 and DU145 cells from migrating through the transwell chambers.

### CMTM5 downregulates EGF-induced receptor signaling and downstream pAkt

To investigate the possible cell signaling pathways involved in EGFR signaling regulated by CMTM5 to decelerate the oncogenicity of PCa cells, we performed western blotting analysis to determine the activities of EGFR and Akt after treatment with EGF. Compared to controls, CMTM5-v1 transfection of PC3 and DU145 cells showed little effect on the total levels of EGFR following EGF stimulation. The EGFR activities, which were indirectly measured through western blotting analysis of p-EGFR^Try1173^, one of the major phosphorylation site in response to EGF, were reduced in cells expressing CMTM5-v1. The phosphorylation of Akt, a key molecule in EGFR downstream signaling, was also decreased in CMTM5-v1-expressing cells. Taken together, these results indicate that ectopic CMTM5-v1 expression could negatively regulate the EGFR/Akt pathway of PC3 and DU145 cells (Fig. 3C).

### CMTM5-v1 promotes the efficacy of the EGFR tyrosine kinase inhibitor Gefitinib

**Figure 4:**
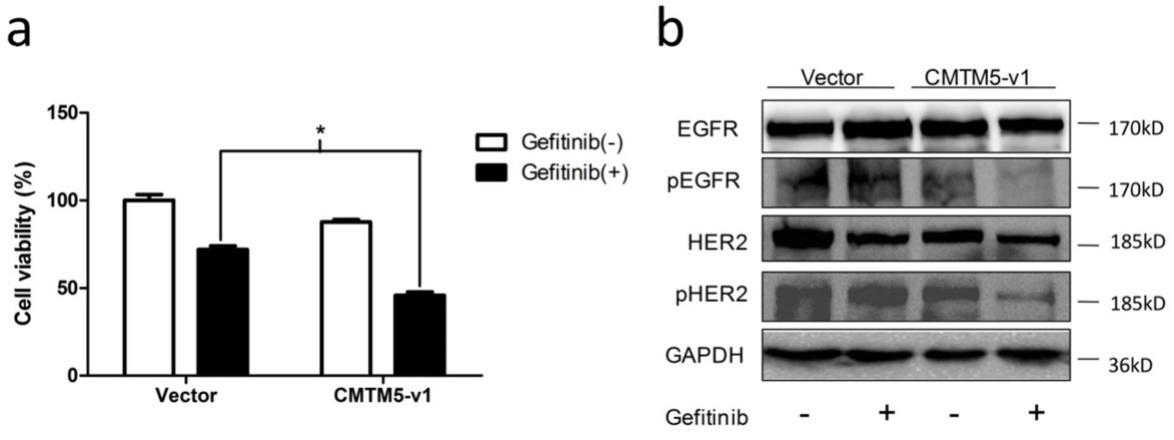
** Effects of CMTM5-v1 on the efficacy of Gefitinib.** (a) Transfected PC3 cells were plated in 96-well plates until adherent and then incubated in normal growth medium supplemented with 10 μM Gefitinib or drug vehicle (0.1% DMSO). Twenty-four hours after treatment, the MTT assay was used to detect cell viability. Data represent the percentages related to vector-transfected cells treated with vehicle. All values were means ± SEM from three independent experiments. (b) Transfected PC3 cells were incubated in normal growth medium with 10 μM Gefitinib or drug vehicle (0.1% DMSO) for 24 h. Whole-cell lysates were immunoblotted with the indicated antibodies.

Restoration of CMTM5-v1 leads to inhibition of EGFR tyrosine kinase activity, which implies that the sensitivity of PCa cells to EGFR TKIs, such as Gefitinib, may be increased by CMTM5. To examine this hypothesis, we treated transfected PC3 cells with Gefitinib or vehicle (0.1% DMSO). As shown in Fig. 4A, the inhibitory effect of Gefitinib on cell viability in cells re-expressing CMTM5-v1 was significantly greater than in cells transfected with vector. To determine whether this synergistic effect of CMTM5-v1 and Gefetinib was attributed to interference with EGFR-related signaling, we evaluated the expression and phosphorylation of EGFR and HER2 in PC3 cells 24 h after incubation in normal growth medium in the presence or absence of 10 μM Gefitinib. Western blot analysis revealed that the p-EGFR levels were decreased in sequence in the four groups with different treatment, and p-EGFR expression was lowest in the cells re-expressing CMTM5-v1 after Gefitinib exposure. However, the overall levels of EGFR did not differ between the groups (Fig. 4C). Following Gefitinib treatment, p-HER2 levels were significantly reduced in the cells expressing ectopic CMTM5-v1 compared with the vector control. Nevertheless, basal HER2 expression seemed to remain the same whether or not the cells expressed CMTM5-v1. These results suggest that CMTM5-v1 enhanced the efficacy of Gefitinib in PC3 cells by decreasing EGFR and HER2 activation.

## Discussion

CMTM5 is a well-characterized tumor suppressor that may related to many types of cancer and to the development and progression of PCa 26, 28-35, 37-39. In this study, we found that CMTM5 expression was significantly downregulated in all the PCa cell lines compared to normal tissues. In contrast, EGFR was overexpressed in PCa cells, especially in the two PCa cell lines, PC3 and DU145, which is consistent with the results in earlier studies 40, 41. Previous studies have shown that CMTM8, another CMTM member containing a MARVEL domain, is associated with the EGFR membrane complex, and the first identified proteins with MARVEL domains could regulate EGFR signaling by accelerating membrane receptor internalization and subsequent degradation in tumor cells 42. In addition, CMTM7 also inhibits cancer cell growth and represses oncogenic EGFR signaling by promoting EGFR internalization and further suppressing the Akt signaling pathway 43. These findings prompted us to assess the functional relevance of CMTM5 and to determine its mechanisms of action in PCa. In the present study, we found that CMTM5-v1 expression inhibited cell proliferation and migration of EGFR-overexpressed DU145, which may be related to suppressed p-EGFR and pAkt expression. Therefore we hypothesized that CMTM5 may be linked to the regulation of transmembrane signaling pathways, especially dysregulated EGFR signaling, which functions as a key phenomenon in sustaining PCa progression and the development of the hormone refractory phenotype.

EGFR is activated after ligand binding and induces a variety of cellular responses, including proliferation, differentiation and migration, mainly through the PI3-kinase/Akt and/or MAPK/ERK pathways 44. We then examined the effects of CMTM5-v1 on the malignant features of PCa cells activated by EGF-induced signaling. We found that ectopic expression of CMTM5-v1 significantly inhibited cell proliferation and chemotactic cell migration in response to EGF. These inhibitory effects in PCa may be due to the negative regulation of EGFR signaling by CMTM5 because phosphorylated EGFR and phosphorylated Akt were also repressed in CMTM5-v1-overexpressing cells after EGF stimulation compared with controls. The results presented in this study are the first demonstration that CMTM5 loss and EGFR deregulation are observed in PCa cells, which provides a crucial signaling platform required for androgen-independent tumor transition and progression and for the malignant behavior of tumor cells.

EGF binding induces receptor homodimerization and activation of tyrosine kinase activity, which results in the initiation of intracellular signal transduction pathways 45. The addition of EGF also induces a rapid internalization of receptors and their degradation after eventually being delivered to lysosomes, which serves as a major negative feedback regulatory mechanism to control the duration and intensity of EGFR signaling 46. Previous studies elucidated that the proteins regulating EGFR endocytosis can also affect downstream signaling activated by EGF 47. For example, exogenous Vav2 expression delays EGFR internalization and stabilizes EGFR at the cell surface, leading to enhanced activity of the EGFR and Akt pathway 48. However, our data revealed that overexpression of CMTM5-v1 hardly decreased total EGFR expression levels, suggesting that the downregulation of EGF-induced signaling by CMTM5 may not be through the reduction of synthesis or the facilitation of specific, direct EGFR degradation. Nevertheless, we thought that CMTM5 might accelerate the ligand-induced removal of EGFR from the cell surface, which is how CMTM8 and CMTM7 regulate EGFR in cancer cells 42, 43. Because internalized receptors are mainly destined for lysosomal degradation and the inactivation of EGFR signaling 49, we hypothesize that CMTM5 might inhibit the oncogenic potential of PCa cells by increasing EGFR internalization and guidance to the lysosomal pathway, which would account for the termination of EGF-induced signaling. Whether CMTM5 exerts this activity and the specific mechanisms involved need to be further explored.

Multiple mechanisms are engaged in the activation of the EGFR pathway during tumor initiation and progression, including receptor amplification and activation of receptor mutations 50. The drugs targeted towards EGFR signaling seem to be of therapeutic relevance in CRPC management 51. However, the results of treatment have been somewhat disappointing 15, 16. This lack of *in vivo* efficacy is also reflected by the high *in vitro* IC_50_ values of Gefitinib for growth inhibition of androgen-independent PC3 and DU145 cells 52. The mutations within the kinase domain responsive to Gefitinib are frequently observed in NSCLC but seldom in PCa 9, 10, 13, 14, 53, 54, which might lead to PCa resistance to TKIs. Considering our result that increased CMTM5-v1 expression reduced EGF- mediated pEGFR^Tyr1173^ levels, one of the tyrosine kinases targeted by TKIs, we tried to test the relationship between CMTM5 and TKIs. Our results revealed that the recovery of CMTM5-v1 expression improved TKI efficacy in PCa cells. HER2 siganling upregulation is observed after treatment with TKIs in CRPC cells or in TKI-resistant cells, making HER2 activation an important factor for the inherent or acquired resistance to EGFR-targeted therapies 55, 56. Our study indicated that after Gefitinib treatment, the EGFR activation and p-HER2 levels were reduced in CMTM5-v1-transfected cells compared to vector-transfected cells. Thus, we also believe that a combination of overexpressing CMTM5 and EGFR-specific TKIs may have a broader effect on EGFR signaling than treatment with TKIs alone, as CMTM5 also inhibited signaling through the EGFR-related receptor HER2. More work is required to assess the utility of the synergistic effects of CMTM5 and TKIs and to establish their detailed mechanism in PCa.

## Conclusions

CMTM5 was downregulated in PCa cells. Ectopic expression of CMTM5-v1 suppresses EGFR- driven PCa cell proliferation and mobility in normal culture conditions and when induced by EGF mainly by inactivating the EGFR/Akt pathway. CMTM5-v1 may promote TKI efficiency and could constitute a new therapeutic strategy for CRPC.
